# Histological Effects of Intravitreal Injection of Antifungal Agents in New Zealand White Rabbits: An Electron Microscopic and Immunohistochemical Study

**DOI:** 10.3390/ph13100267

**Published:** 2020-09-23

**Authors:** Sofia Karachrysafi, Antonia Sioga, Anastasia Komnenou, Athanasios Karamitsos, Maria Xioteli, Ioanna Dori, Georgios Delis, Evangelia Kofidou, Penelope Anastasiadou, Sotiris Sotiriou, Vasileios Karampatakis, Theodora Papamitsou

**Affiliations:** 1 Laboratory of Histology-Embryology, School of Medicine, Faculty of Health Sciences, Aristotle University of Thessaloniki, 54124 Thessaloniki, Greece; sioga@auth.gr (A.S.); thpapami@auth.gr (T.P.); 2 School of Veterinary Medicine, Faculty of Health Sciences, Aristotle University of Thessaloniki, 54124 Thessaloniki, Greece; natakomn@vet.auth.gr (A.K.); evikofidou@gmail.com (E.K.); 3 2nd University Eye Clinic, Papageorgiou Hospital, Aristotle University of Thessaloniki, Greece: Ring Road, Nea Efkarpia, 56403 Thessaloniki, Greece; karamitsos_thanos@yahoo.com; 4 Laboratory of Anatomy, Histology and Embryology, School of Veterinary Medicine, Faculty of Health Sciences, Aristotle University of Thessaloniki, 54124 Thessaloniki, Greece; mchiotel@vet.auth.gr (M.X.); idori@vet.auth.gr (I.D.); 5 Laboratory of Pharmacology, School of Veterinary Medicine, Faculty of Health Sciences, Aristotle University of Thessaloniki, 54124 Thessaloniki, Greece; delis@vet.auth.gr; 6 Department of Oral Medicine/Pathology, School of Dentistry, Faculty of Health Sciences, Aristotle University of Thessaloniki, 54124 Thessaloniki, Greece; panastasiadoy@yahoo.gr; 7 Department of Laboratory Medicine and Pathology, Mayo Clinic, Rochester, MN 55905, USA; sotiris_sot@hotmail.com; 8 Laboratory of Experimental Ophthalmology, School of Medicine, Faculty of Health Sciences, Aristotle University of Thessaloniki, 54124 Thessaloniki, Greece; karophth@auth.gr

**Keywords:** voriconazole, micafungin, intravitreal injection, retina, histology, TNF-a, IL-6, electron microscopy

## Abstract

Fungal endophthalmitis is a serious and vision-threatening infection which requires an immediate and effective treatment approach. Our research aims to elucidate the histological effects of the intravitreal injection of the maximum safe dosage of voriconazole and micafungin on retina. Six albino New Zealand White Rabbits were used. In experimental animals, a solution of voriconazole (Group V) or micafungin (Group M) was intravitreally injected in the right eye, while in control animals, balanced salt solution was intravitreally injected in the left eye (Group C). Euthanasia was performed ten days post injection and the retina was removed and prepared for histological examination with a light and electron microscope. Eosin-hematoxylin staining did not reveal any pathological changes in any of the samples examined. The immunohistochemical staining for Tumor Necrosis Factor alpha (TNF-a) marker was detected as negative in all samples, while Interleukin 6 (IL-6) marker was detected as mild only in the group injected with voriconazole. Electron microscopy revealed several ultrastructural alterations in retinal layers in both groups of experimental animals. Histological retinal lesions, revealed with electron microscopy in the present investigation, raises the question of the safe usage of these antifungal agents in the treatment of fungal intraocular infections in the future.

## 1. Introduction

Fungal endophthalmitis is a serious and vision-threatening infection with increasing incidence during the last decades. Various factors, such as the use of corticosteroids, which facilitates the penetration of pathogens, and the spread of topical antibiotics, which create an environment of lower competition between microorganisms on the surface of the eye, are suggested as key factors for this increase [1]. Fungal endophthalmitis is a life-threatening infection, as well as a common complication of intraocular surgery (mainly after cataract surgery), the use of ophthalmic products and/or corticosteroids, and pharmaceuticals (mainly drugs), eye injuries, systemic fungal infections (mainly candidiasis and aspergillosis), and immunosuppressive entities (mainly Human Immunodeficiency Virus (HIV) infection) [2,3,4,5,6,7,8,9,10,11,12]. Fungal infections usually affect the cornea (fungal keratitis), the vitreous (fungal endophthalmitis), and ocular tunics, such as sclerosis (fungal panophthalmitis). The most common organisms found in fungal endophthalmitis belong to the species *Candida*, *Aspergillus* and *Fusarium* [2,4,13].

Despite the emergence of new drugs, treatment is still difficult in many cases. Compared to antibacterials, antifungal drugs have lower efficacy due to their mechanism of action (usually antifungal agents with dose-dependent fungicidal action), lower tissue penetration, and less noisy nature of the infection [14].

Intravitreal administration of antifungal agents is one of the dominant therapeutic approaches in these ocular diseases. In the past, amphotericin B (AMB) was the only antifungal agent approved for intravitreal administration. However, the retinal necrosis caused by low drug levels as well as the development of resistance to its action by many types of fungi have necessitated the use of other antifungal agents with fewer side effects and possibly higher efficacy in the treatment of fungal endophthalmitis [15].

The newest representatives investigated in the present study are voriconazole, which belongs to the azoles, and micafungin, which belongs to the echinocandins.

Voriconazole is a second generation triazole which acts on the enzymes of the fungal cytochrome P450, preventing the synthesis of ergosterol in the cytoplasmic membrane, thus inhibiting fungal growth [1,14,16,17]. It has shown therapeutic efficacy against fungal keratitis and endophthalmitis after intrastromal, intracameral, intravitreal, intravenous, oral, and topical administration [18,19,20,21,22,23,24]. It has strong biological activity against *Candida*, *Aspergillus*, *Fusarium,* and other filamentous fungal species [25,26,27]. Voriconazole is metabolized in the liver (a feature that promotes its hepatotoxicity), while it also induces vision disorders (usually reversible), rare skin rashes, and teratogenicity, as additional side effects [1,14,17,27].

Micafungin acts by inhibiting the synthesis of *β*-(1,3)-D-glucan, which is an important structural element that maintains the integrity of the fungal cell wall. It presents fungicidal action against various species of *Candida* and fungistatic action against *Aspergillus* [28,29,30]. In clinical practice it is administered mainly intravenously or topically. Intravitreal administration has only been attempted in experimental protocols with laboratory animals. Its safety profile has made micafungin a very promising antifungal agent [1,2].

In our protocol, we studied two immunological markers, IL-6 and TNF-a, in order to clarify the possible presence of inflammation in retina.

IL-6 is a cytokine produced by various cells such as T- and B-lymphocytes, monocytes/macrophages, neutrophils, fibroblasts, endothelial cells, hepatocytes, mesangial cells as well as cancer cells [31,32]. It plays a dual role in the eyes. On the one hand, IL-6 protects ocular tissues from harmful infections and on the other hand, it can damage sensitive ocular elements through undesirable neovascularization or recrudescence of inflammation [33].

TNF-a is an important pro-inflammatory cytokine with pleiotropic functions composed primarily by T-lymphocytes and macrophages/monocytes and to a lesser extent by neutrophils and mast cells [34,35]. during acute inflammation leading to necrosis or apoptosis. It is also important in resistance to infection and cancer [34,36,37,38,39,40,41,42]. TNF-a is involved in the pathogenesis of many ocular inflammatory diseases [34].

To our knowledge, there are to date no similar histological and immunohistochemical studies of the impact of the above drugs after intravitreal injection. Our research aims to elucidate the histological effects of the intravitreal injection of the maximum safe dosage of voriconazole and micafungin according to available literature on retina.

## 2. Results

### 2.1. Group C

**Figure 1:**
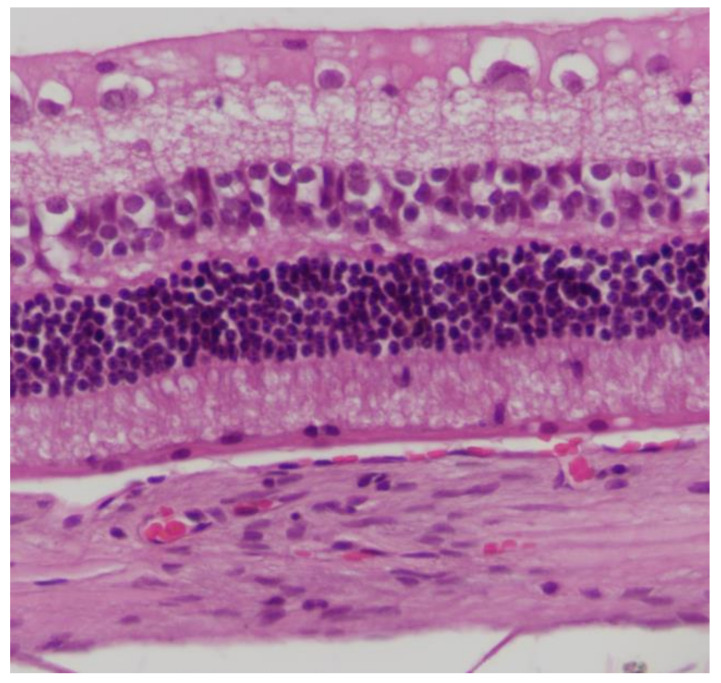
***Group C.*** No pathological changes were observed on retina. EH ×200.

**Figure 2:**
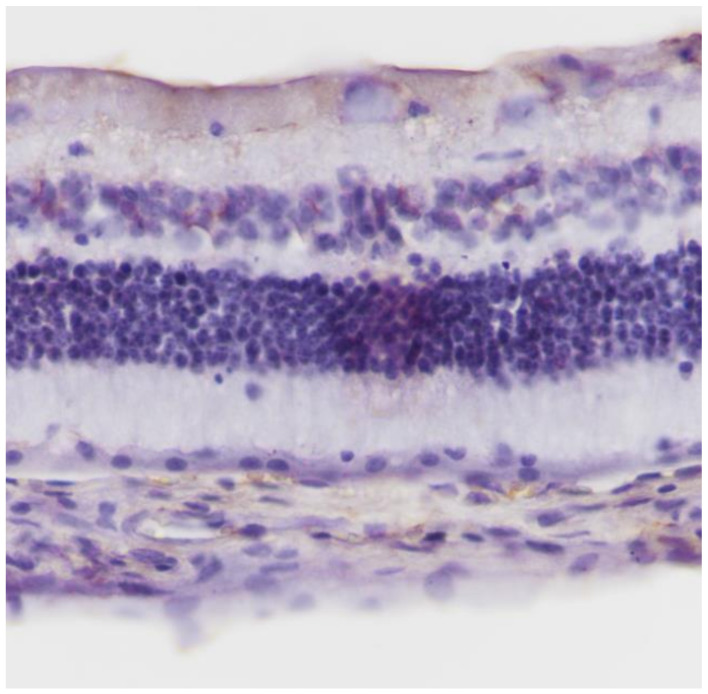
***Group C.*** Negative (−) immunohistochemical staining.IL-6 ×200.

**Figure 3:**
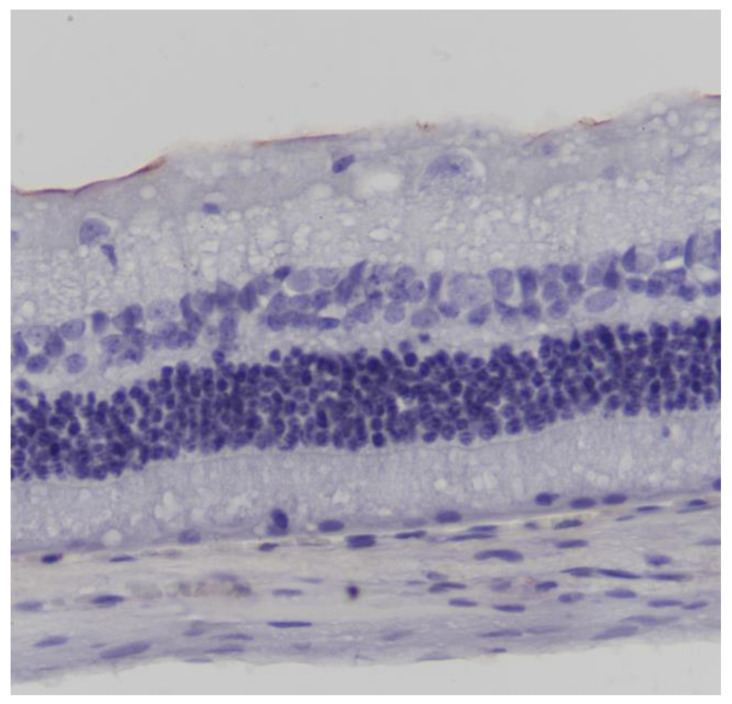
***Group C.*** Negative (-) immunohistochemical staining. TNF-a ×200.

**Figure 4:**
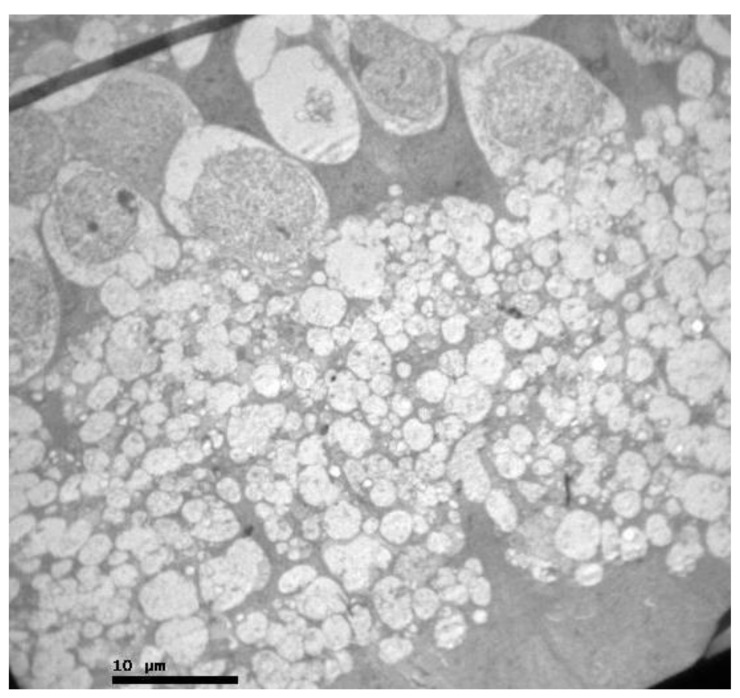
***Group C.*** Nerve fiber and ganglion cell layer with no ultrastructural pathological alterations.

**Figure 5:**
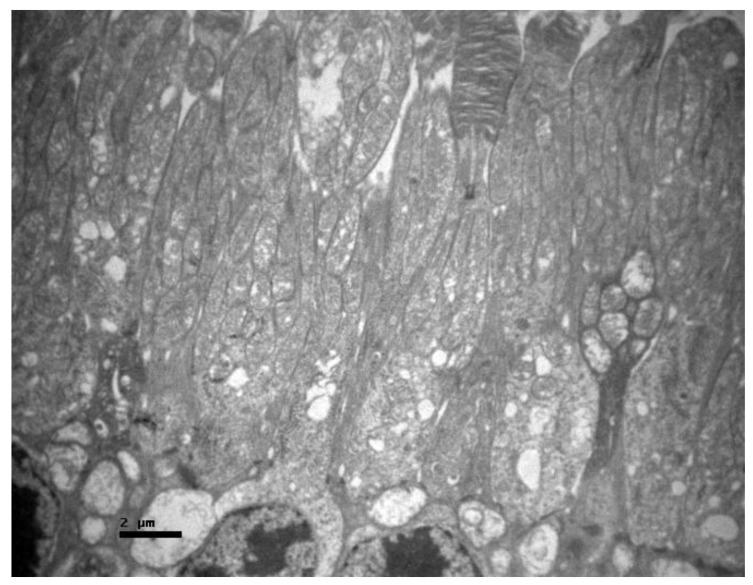
***Group C.*** Photoreceptor layer with no ultrastructural pathological alterations.

No macroscopic lesions were found in all samples. Light microscopic examination of eosin-hematoxylin stained sections did not reveal any histopathological changes (Figure 1), while the immunohistochemical stainings for TNF-a as well as IL-6 markers were found to be negative (−) in all samples examined (Figure 2 and Figure 3). Electron microscopy did not reveal any ultrastructural pathological alterations in the retina in all samples (Figure 4 and Figure 5).

### 2.2. Group V

**Figure 6:**
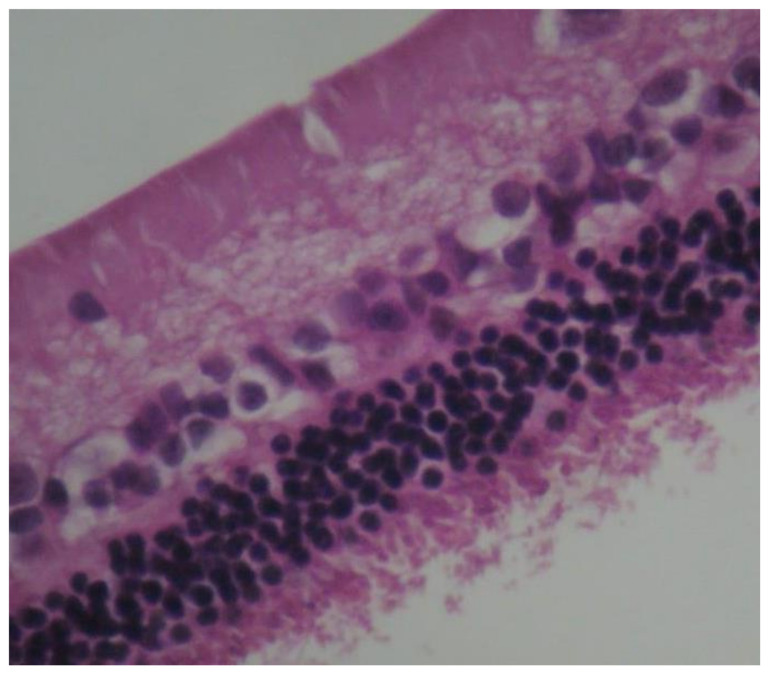
***Group V.*** No pathological changes were observed in the retina. EH. ×240.

**Figure 7:**
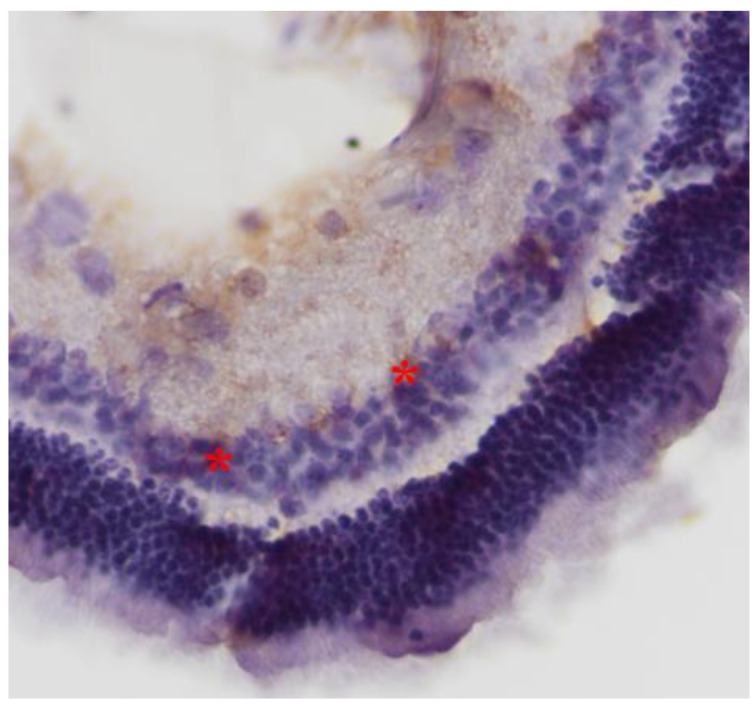
***Group V.*** Mild intensity (+) of immunohistochemical staining. Presence of lymphocytes (*). IL-6. ×200.

**Figure 8:**
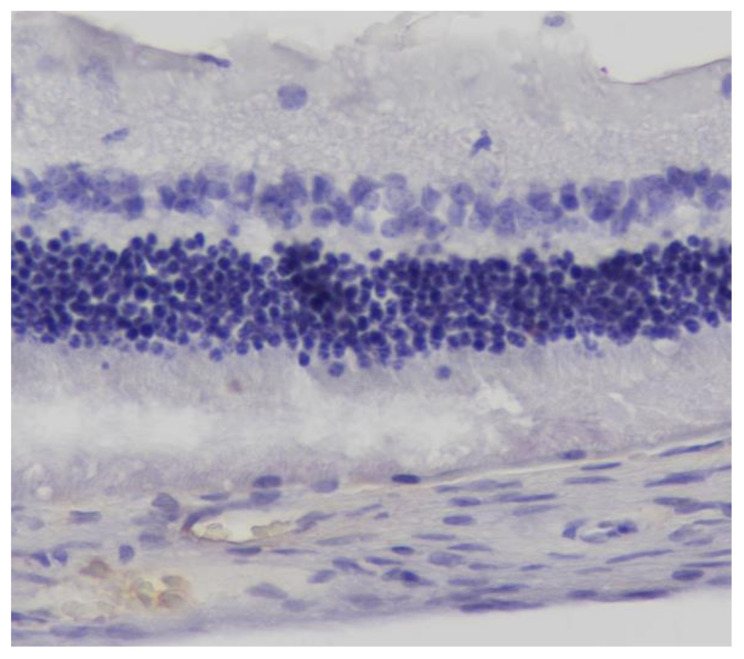
***Group V.*** Negative (−) immunohistochemical staining. TNF-a. ×200.

**Figure 9:**
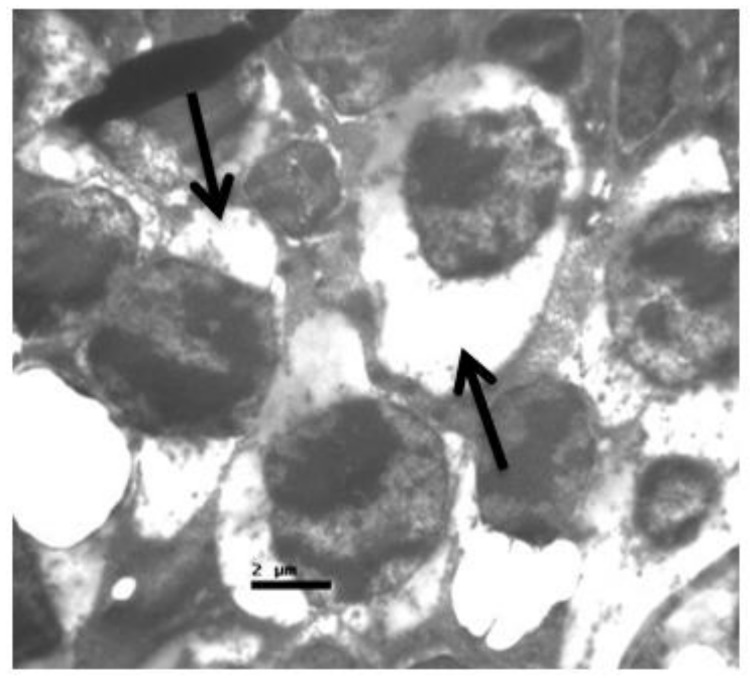
***Group V.*** Rods and cones with sparse cytoplasm around the nucleus in the outer nuclear layer. (↑).

**Figure 10:**
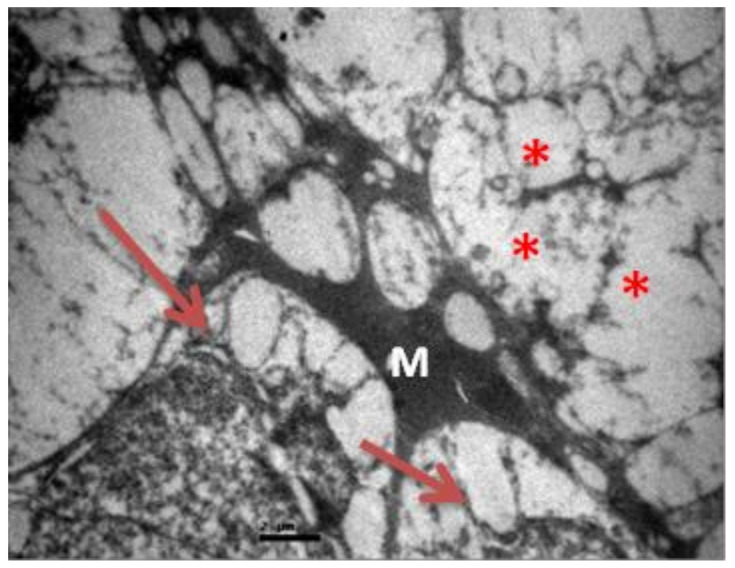
***Group V.*** Loss of the organized structure of neurofibrils and microtubules. (*) Müller cell.(M)Ganglion cells with vacuolization and dilatation of the rough endoplasmic reticulum. (↑).

**Figure 11:**
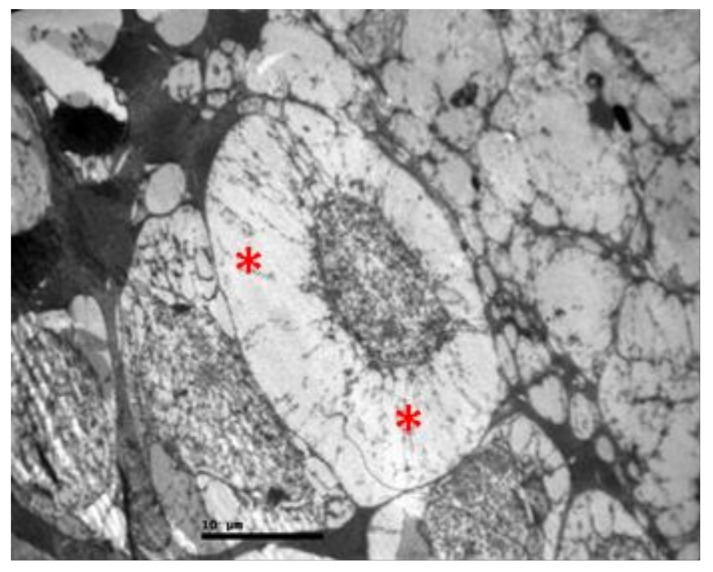
***Group V.*** Ganglion cell with vacuolization of the cytoplasm and dilatation of the rough endoplasmic reticulum in the ganglion cell layer. (*).

No macroscopic lesions were found in all samples. Light microscopic examination of eosin-hematoxylin stained sections did not reveal any histopathological changes in all samples (Figure 6). The immunohistochemical staining for the IL-6 marker was determined as mild (+) with the presence of lymphocytes, in all samples (significant difference between group V and all other groups) (Figure 7). On the other hand, staining for TNF-a marker was found negative (−), in all samples (significant difference from IL-6 within the group) (Figure 8). Electron microscopy revealed rods and cones with sparse cytoplasm around the nucleus in the outer nuclear layer (Figure 9). In the nerve fiber layer, loss of the organized structure of neurofibrils and microtubules in addition to merging of the unmyelinated nerve fibers were observed. Ganglion cells presented vacuolization as well as dilatation of the rough endoplasmic reticulum in the ganglion cell layer (Figure 10 and Figure 11). The ultrastructural lesions were observed in all samples.

### 2.3. Group M

**Figure 12:**
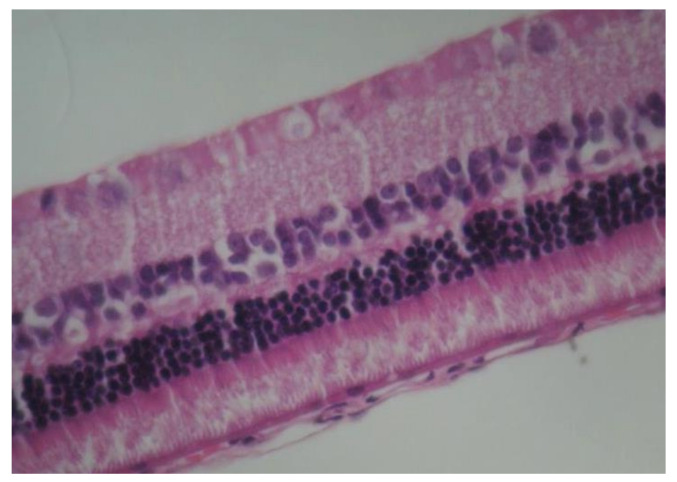
***Group M.*** No pathological changes were observed in the retina. EH. ×160.

**Figure 13:**
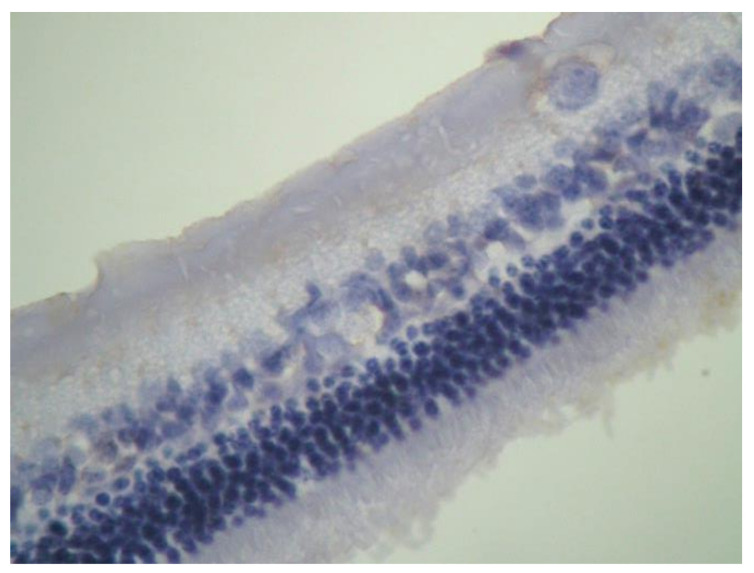
***Group M.*** Negative (−) immunohistochemical staining. IL-6. ×160.

**Figure 14:**
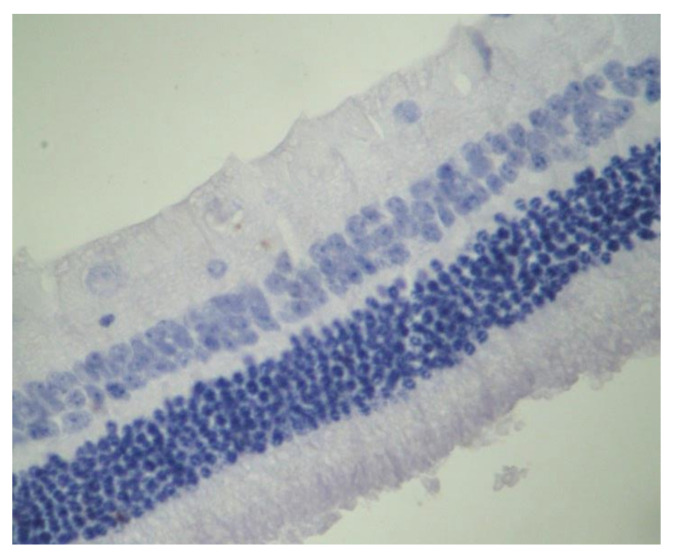
***Group M.*** Negative (−) immunohistochemical staining. TNF-a. ×160.

**Figure 15:**
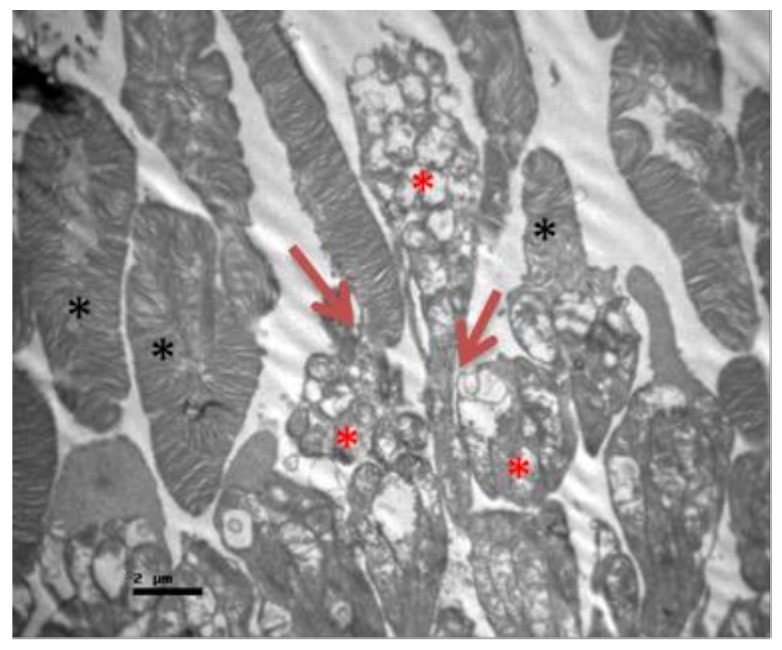
***Group M.*** Separation between the outer and inner segment and absence of the connecting cilium of the photoreceptors. (↑) In the outer segment, the membranes are separated in the middle leaving a gap and are not parallel to each other. (*) Εnlarged mitochondria in the inner segment with minimal folds (*).

**Figure 16:**
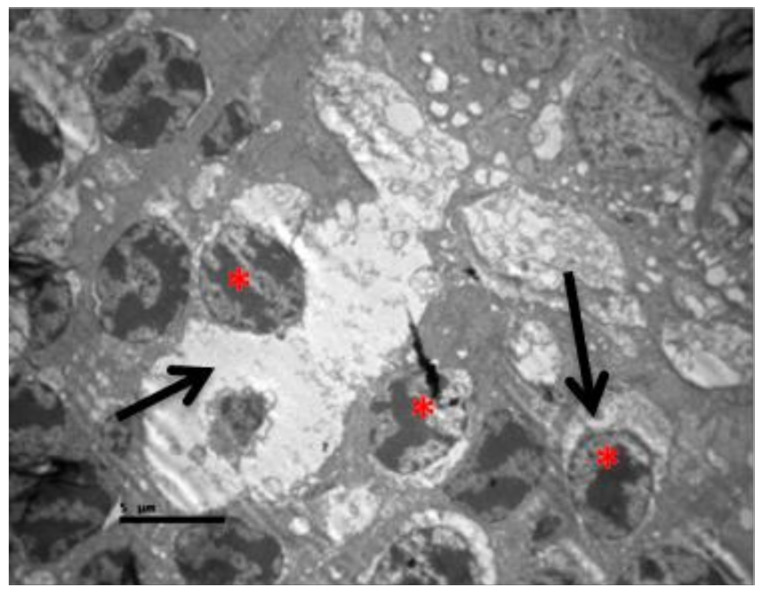
***Group M.*** Degeneration of some photoreceptors’ nuclei (*) and vacuoles in the intercellular space (↑).

**Figure 17:**
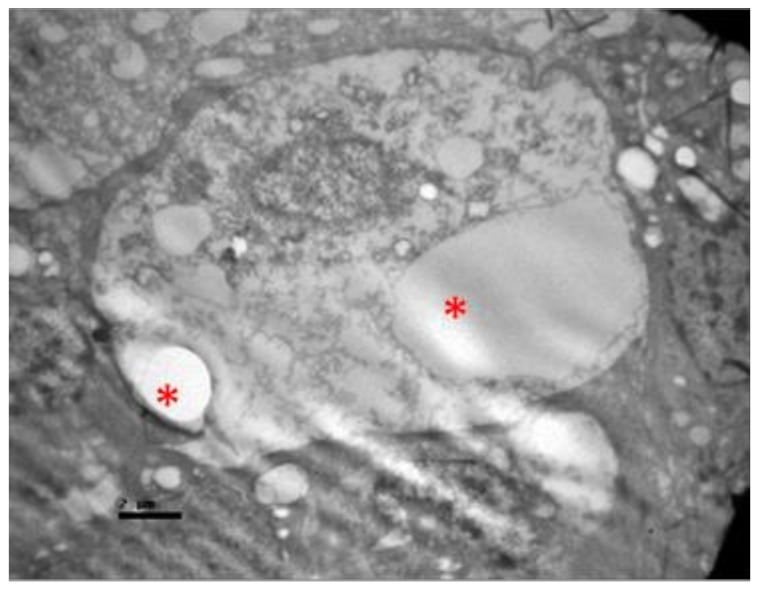
***Group M.*** Ganglion cells with enlarged vacuoles (*) in the cytoplasm and only few organelles.

Eosin-hematoxylin staining did not reveal any pathological changes in all samples (Figure 12). Regarding the immunohistochemical stainings for IL-6 as well as TNF-a markers, they were detected as negative (−) in all samples (Figure 13 and Figure 14). Electron microscopy revealed a pathological structure of the outer part of the inner segment of the photoreceptors in the photoreceptor layer. Separation between the outer and inner segment and the absence of the connecting cilium of the photoreceptors was observed. In the outer segment, the membranes are separated in the middle leaving a gap and are not parallel to each other. In addition, the mitochondria in the inner segment were enlarged with minimal folds. (Figure 15). Also, some nuclei of the photoreceptors presented degeneration in the outer nuclear layer with vacuoles in the intercellular space (Figure 16). In the ganglion cell layer, ganglion cells presented a vacuolated cytoplasm with only few organelles (Figure 17). The ultrastructural lesions were observed in all samples.

## 3. Discussion

Voriconazole is an antifungal agent belonging to the triazole family and has been widely used in the treatment of ocular fungal infections through topical, oral and intraocular route of administration. Several investigations have been conducted to study its mode of action, activity against various fungal species, bioavailability and pharmacokinetics, as well as its toxicity with related side effects.

It has been reported, that voriconazole concentrations in vitreous and aqueous humor accounted for 38% and 51% of plasma levels of the substance, respectively, after oral administration. Although the concentrations achieved in vitreous were not sufficient in order to treat infections caused by *Fusarium spp.*, the authors argue that the study was performed on non-inflamed eyes and that, in the presence of inflammation, a more permeable blood–retinal barrier would contribute to the increase of drug’s topical concentration [43].

Other investigators recommended voriconazole as the drug of choice for oral use in the treatment of deep keratitis, scleritis and endophthalmitis as well as the prophylaxis after penetrating keratoplasty [44]. Oral administration of voriconazole as a precaution in case of ocular injury by organic material has also been recommended [45].

There are also many reports of therapeutic success with topical administration of the drug. Administration at a concentration of 1 mg/mL was effective in the treatment of keratitis *from Candida, Aspergillus, Fusarium, Scedosporium,* and *Paecilomyces*, among others [22,46,47,48,49].

Various studies in horses have shown drug penetration even with epithelial integrity [50]. Some reports support the intracorneal use of voriconazole in cases of deep keratitis that does not respond to topical and / or oral administration. Prakash and colleagues report success in three cases of keratitis that did not respond to the topical administration of natamycin, using voriconazole at a dose of 50 μg/0.1 mL [51]. Recently, three cases of *Fusarium* keratitis, have been described that did not respond to topical therapy, but were treated with intracorneal administration of voriconazole [52]. Sharma and colleagues, in a series of 13 patients, also recommend the use of intrastromal voriconazole in resistant keratitis [21].

It has been suggested that direct injection of voriconazole into the cornea increases its concentration above the minimum inhibitory concentration (MIC) for *Fusarium* species. However, there are few studies comparing voriconazole with other antifungal agents. In a multicenter randomized study, voriconazole was not found to be superior to natamycin, and both groups showed similar healing times and final visual acuity [53].

There have even been reports of treatment failure with voriconazole. Giaconi and colleagues reported two cases, one of keratitis due to *Fusarium oxysporum* and another due to *Colletotrichum dematium*, that did not respond to topical drug therapy [54]. In vitro studies demonstrate the superiority of voriconazole over amphotericin B against *Aspergillus spp* [55,56,57,58]. Against *Fusarium* species, the absolute MICs of voriconazole, natamycin and amphotericin B were similar while voriconazole has a lower relative MIC than polyenes [29]. However, the minimum inhibitory concentration of voriconazole for *Fusarium* was higher than that for *Candida* and *Aspergillus* [59].

In addition to the above investigations, two experimental studies have been published regarding the effect of intravitreal injection of voriconazole on the retina.

Particularly, Gao et al. demonstrated that intravitreal administration of voriconazole to rats at an intravitreal concentration of up to 25 μg/mL did not cause electroretinographical or histological lesions (study with eosin-hematoxylin staining). Specifically, the parameters that were statistically compared were b- and a-wave in the ERG, while no statistical analysis was performed on the histological findings. The aforementioned intravitreal concentration in the human eye corresponds to a dose of 100 μg and does not cause long-term lesions. At doses higher than those mentioned above, voriconazole may cause retinal necrosis. Therefore, voriconazole is considered safer to use than amphotericin B in intravitreal infusion. This protocol was applied by administering only one intravitreal injection and not multiple [6].

Also, Harrison, Glickman, and colleagues compared the intravitreal administration of amphotericin B, voriconazole, and micafungin to rabbit eyes by studying the electro-retinography and histological lesions by applying eosin-hematoxylin staining. They concluded that amphotericin B and micafungin are equally effective in maintaining retinal function in the first 72 h after administration. However, micafugin is less toxic. On the other hand, voriconazole has a disadvantage in maintaining retinal function compared to the other two substances, requires higher concentrations, and is considered more toxic than micafungin. Also, this protocol refers to the administration of only one intravitreal injection and not multiple [60].

Therefore, there is limited data on the effect of voriconazole on the retina after administration of either single or multiple intravitreal injections regarding the histological findings as well as the parameters of inflammation. Most studies focus either on the therapeutic capacity of the drug depending on the route of administration and the type of fungus or on its pharmacokinetics and bioavailability. There are also many clinical cases that report the empirical application of voriconazole in the form of either single or multiple intravitreal injections but none of them document either histologically or through ERG its effect on the retina.

Micafungin has also been evaluated for its efficacy and safety in ocular fungal infections. Studies have been performed both in vivo (animal models) and in clinical settings (human study objects). The first studies have been performed on rabbit models. In the study by Trujillo et al., the topical application of micafungin was compared with the topical application of natamycin and saline (control group) in the treatment of *Aspergillus* keratitis in rabbits without a significant difference in treatment with natamycin and micafugin [61]. In addition, treatment was found to be well tolerated demonstrating the safety and efficacy of micafungin in the treatment of fungal keratitis.

In a study by Paris et al., the efficacy of intravitreal and intravenous (IV) administration of micafungin was compared with that of intravitreal and IV administration of amphotericin B and saline (control group) in the treatment of *Aspergillus* keratitis [62]. Evaluation was performed by recording ERGs, while aqueous humor and vitreous were analyzed after sacrifice to quantify the level of the content antifungal drug. It was observed that both drugs reduced the fungal load and maintained the range of ERG, indicating the absence of any detrimental effect on retinal cells. In addition, quantification revealed the presence of micafungin in infected eyes for several days in the case of intravitreal administration.

In a similar study by Harrison et al., intravitreal injection of micafungin showed similar results to standard treatment with voriconazole and amphotericin B in *A. Fumigatus* keratitis, which was demonstrated by the maintenance of b-wave length in the ERG for all three antifungal therapies compared to the control group that received saline [60]. According to Hiraoka et al., topical application of micafungin (0.1%) was evaluated against the control group in uninfected rabbit eyes [63]. The eyes were assessed for corneal thickness, endothelial cell density, intraocular pressure, and lacrimal lactate dehydrogenase activity. It was observed that all evaluated parameters, except corneal thickness, did not differ significantly from the control group. The corneal thickness, however, was significantly smaller in the micafungin group, with the thickness being restored within 24 h after final application. Histopathological studies of the cornea did not reveal any toxicity to the cornea, enhancing further the safety and tolerability of micafungin in the eye.

The ocular distribution of micafungin was estimated by Suzuki and colleagues in rabbit eyes after intravenous administration [64]. Plasma, choroid, retina and vitreous analysis were performed in order to assess micafungin content in each tissue. Micafungin was detected at levels above its MIC in plasma, choroid and retina, but was not detected in vitreous, suggesting the potential utility of the drug in choroidal and retinal fungal infections.

There are, of course, several studies that have reported the evaluation of micafungin in human patients. Toshikuni and colleagues reported that co-administration of micafungin with fluconazole eye drops was proved to be more effective in treating *C. albicans* endophthalmitis than systemic fluconazole treatment in a patient with cirrhosis of the liver [65].

Endogenous endophthalmitis due to *Trichosporon* species has also been shown to be treated effectively and safely within six weeks with co-administration of voriconazole and micafungin in a patient with diffuse trichosporonosis [66]. The efficacy and safety of topical micafungin therapy (0.1%) were found to be similar to topical fluconazole therapy (0.2%) in the treatment of *Candida* keratitis [67]. No statistically significant differences in clinical outcomes were observed in either treatment, such as the healing period, the opacity and permeability of the cornea, the restoration of visual acuity indicating non-superiority of micafungin therapy compared to standard fluconazole therapy in fungal keratitis. Micafungin in intravenous administration to a patient suffering from *C. albicans*-induced endophthalmitis showed drug penetration into vitreous and aqueous humor. However, only in the vitreous, the concentration of micafungin was above its MIC for *C. albicans* [68].

In another study of the same research team, it was observed that intravenous administration of micafungin in endogenous endophthalmitis showed low levels of the drug (less than MIC) in the aqueous humor and vitreous. These differences were attributed to various changes in blood-retinal barrier’s integrity in the 2 studies due to differences in the severity of inflammation. These differences indicate the need for concomitant intravitreal infusion of other antifungal agents in combination with intravenous micafungin therapy [69]. Intravenous administration of micafungin, however, resulted in concentrations higher than its MIC in the cornea, choroid, and retina, suggesting a potential therapeutic role in the treatment of fungal infections at these sites.

In another study by Mochizuki et al., a clinical failure of intravenous micafungin (200 mg/day) in the treatment of *C. tropicalis* endophthalmitis was observed [70]. β-(1,3)-D-glucan levels were used to assess the reduction of symptoms during antifungal therapy. Due to the failure of fluconazole, itraconazole and levofloxacin to reduce the level of β-(1,3)-D-glucan, micafungin therapy was started. However, even after micafungin treatment, β-(1,3)-D-glucan continued to rise indicating treatment failure. This failure was in contrast to the in vitro sensitivity of *C. tropicalis* to micafungin at a MIC of 0.03 mg/mL, which was determined simultaneously. This clinical failure was attributed by the authors to the characteristic paradoxical phenomenon exhibited by echinocandins [71]. However, this hypothesis was not examined by the authors, and therefore remains a conjecture.

In a clinical case reported by Monden et al., topical and intravenous administration of micafungin was found to be effective and safe in the treatment of fungal keratitis due to *Pestalotiopsis clavispora* after prior treatment with topical voriconazole and pimaricine in recurrence of infection [72]. Micafungin in combination with voriconazole has been shown to be effective in treating fungal keratitis caused by *Beauveria bassiana* when treatment with topical and systemic voriconazole and topical natamycin has not yielded favorable clinical results [73]. Treatment success was attributed to the synergistic effect of voriconazole and micafungin and surgical clearance. Thus, the inclusion of micafungin in the treatment helped to better manage keratitis. The synergistic effect of voriconazole and micafungin has also been used in the treatment of postoperative endophthalmitis caused by *Aspergillus tubingenesis* [74]. Topical micafungin has been shown to be effective and safe in treating fungal keratitis caused by *Wickerhamomyces anomalus* [75]. as well as in treating fungal keratitis and endophthalmitis caused by various fungal species and could be considered first-line antifungal agent in the treatment of ocular fungal infections [2].

It is obvious that most studies refer to the clinical features of drug use and especially in cases where intraocular fungal infection has been induced. Most experimental protocols study the induction of fungal intraocular infection and the therapeutic response to different doses of antifungal agents. There is also a lack of data on the histological effects of drugs on the retina without a history of fungal infection, with the exception of a study by Harrison et al. in which a histological study with eosin-hematoxylin staining in rabbits’ eyes was performed after voriconazole as well as micafungin administration. However, histological lesions were observed only in the eyes in which the drug was administered in combination with fungal infection, while in the eyes where the drug was only injected no lesions were observed. Regarding the dosages, intravitreal injection of micafungin at a dose of 0.06 mL containing 15 μg of micafungin and injection of voriconazole at a dose of 0.06 mL containing 150 μg of voriconazole were performed [60].

Also, a study by Gao et al. in rat eyes showed that intravitreal administration of voriconazole in order to achieve an intravitreal concentration of 5–25 µg/mL did not cause retinal lesions, while intravitreal concentration of 50 to 500 μg/mL caused small foci of retinal necrosis, with disorganization especially of the photoreceptor layer and the inner nuclear layer, as well as degeneration of the photoreceptors. In contrast, the ganglion cell layer remained intact. At an intravitreal concentration of more than 500 μg/mL voriconazole caused more focal necrotic areas in the retina with more pronounced photoreceptor degeneration and disorganization of the photoreceptor layer and the inner nuclear layer. In fact, focal detachment of the retina was observed in these necrotic areas. It is noteworthy that inflammatory cells were also observed in these focal areas of the retina in the presence of choroidal congestion. Therefore, Gao and his colleagues recommend intravitreal voriconazole concentration up to 25 mg/mL as safe [5]. Gao’s findings are consistent with our own findings, as for the same intravitreal voriconazole concentration of 25 mg/mL, eosin-hematoxylin staining showed no morphological retinal lesions.

Regarding to micafungin, according to Paris and colleagues, intravitreal administration of micafungin at a dose of 150 μg to rabbit eyes does not cause ERG lesions [62]. However, ERG was the only method of detecting retinal damage, as no histological analysis was performed. According to Kapur, intravitreal administration of micafungin to rabbit eyes at a dose of up to 0.025 mg/0.1 mL did not cause histopathological lesions or ERG lesions, suggesting this dose as a safe non-toxic starting dose with adequate antimicrobial action and therefore for future use in humans for the treatment of fungal endophthalmitis [76]. However, Kapur’s findings are consistent with our findings, as for the same dose of 0.025 mg/0.1 mL micafungin, eosin-hematoxylin staining showed no morphological changes in the retina.

Finally, there are to date no reports on the study of IL-6 and TNF-α in the retina after injection of voriconazole and micafungin, which further enhances the originality of this research work. Immunohistochemical stainings were negative for TNF-α in both voriconazole-infused and micafungin-infused eyes, which suggests the absence of inflammation and implies that TNF-α is not involved in the mechanism of retinal damage. In contrast, immunohistochemical staining for IL-6, while was detected as negative for ocular injection of micafungin, was detected as mildly positive for ocular injection of voriconazole, demonstrating its potential pro-inflammatory role in the mechanism of retinal lesion after infusion.

Regarding the electron microscopy, no previous studies have been conducted in order to clarify the possible ultrastructural retinal lesions after voriconazole and micafungin injection intravitreally. To our knowledge, the present study it is the first to demonstrate ultrastructural lesions in the retina following voriconazole as well as micafungin injections. While eosin-hematoxylin staining did not reveal any lesions, electron microscopy sheds light into the morphological alterations of the nerve fibers, and the cytoarchitecture of ganglion and photoreceptor layers indicating a possible toxic action of the previously considered safe dosages of these antifungal drugs.

## 4. Materials and Methods

### 4.1. Materials and Animals

A total of six (6) New Zealand White Rabbits (2–4 kg body weight), aged five (5) months, were used, including male and female animals. Both eyes of each rabbit were used. New Zealand White Rabbits were used due to the similarity of their ocular tissues to human ocular tissues as previous protocols have described. The number was limited to six in order to respect the principles of the 3Rs (Replacement, Reduction and Refinement) that were developed over 50 years ago providing a framework for performing more humane animal research.

Solutions of micafungin (Mycamine, Astellas Pharma Europe B.V.), voriconazole (VFend, Pfizer Europe MA EEIG), or Balanced Salt Solution (BSS) (IOLART, Greece) were injected into the mid-vitreous of the rabbits’ eyes. Considering that the rabbit’s average vitreous volume is approximately 1.6 mL [4,5,60,77,78]., voriconazole was administered at a dose 40 μg/0.1 mL in order to achieve an intravitreal concentration of 25 μg/mL. Micafungin was administered at a dose of 25 μg/0.1 mL. BSS was administered at a dose of 0.1 mL.

The eyes of animals were assigned to three groups: Group V, consisted of the right eyes of three rabbits in which a single intravitreal injection of voriconazole was administered on day 0. Group M, consisted of the right eyes of three rabbits in which a single intravitreal injection of micafungin was administered on day 0. Group C, comprised the left eyes of all six rabbits in which one intravitreal injection of BSS was administered on day 0. Groups V and M were the study groups, while Group C was used as the control group.

The rabbits were housed in specially designed cages, made of carefully selected materials that do not affect the animals’ health while also providing easy access for everyday cleanliness and care. That included unlimited access to fresh, clean water, condensed aliment and hay, which were daily renewed. The cages were equipped with a ventilation system, in order to maintain stable temperature, and with a lightning regulation system.

### 4.2. Methods

#### 4.2.1. Procedures

The rabbits were weighed and anesthetized using dexmedetomidine 50 μg/kg and butorphanol 0.1 mg/kg (i.m.) for prenarcosis as well as ketamine 25 mg/kg i.m. and propofol 0.5 mg/kg i.v. (whenever additional time for deep anaesthesia was required). The surface of the eye was anesthetized with proparacaine hydrochloride 0.5%. The mydriasis of the pupils was induced by tropicamide 1%. Voriconazole, micafungin and BSS were administered to the center of the vitreal body via a 30 Gauge needle. After the injection, a cotton patch was placed over the site for 30 s to prevent any leakage. Ophthalmological examination under a slit lamp and indirect ophthalmoscopy were performed to all rabbits prior to sacrifice. Euthanasia of all animals was performed on day 10 post injection with the use of dexmedetomidine, ketamine, propofol and KCl. Subsequently, the retina from each eye was removed, and retinal samples were obtained. The specimens were then prepared for light microscopy and immunohistochemical staining.

#### 4.2.2. Preparation for Light Microscopy and Immunohistochemistry

Specimens were dissected into tissue blocks of 0.5 to 4.0 cm thickness and put into cassettes. They were then fixed by immersion into a 10% formalin solution (out of 35% formaldehyde stock solution). Subsequently, they were dehydrated through an ascending series of alcohol solutions (76%, 96%, 100%, 100%). Tissues were cleared into xylene for four hours. They were then dipped into liquid paraffin for additional four hours, so that embedding could follow. This was performed by placing the specimens into metallic molds, soaking them into liquid paraffin and allowing them to cool in 4 °C for twenty minutes.

Thereupon, sectioning of the paraffin blocks was performed, using a semi-automated microtome, at a thickness of 3 μm. Ten saggital sections were collected by systematic random sampling. Three of these sections were placed on standard microscope slides, whilst the other seven were placed on seven positively charged slides. They were all allowed to dry at room temperature for one hour. The first two sections, destined for morphological analysis, were placed in the oven for one hour at 65 °C and deparaffination followed by dipping them in xylene solution for ten minutes. They were then hydrated through a descending series of alcohol solutions (100%, 100%, 96%, 76%). Sections were then stained with hematoxylin for five minutes and rinsed in tap water for five more minutes. For the partial discoloration of hematoxylin, a 1% differentiation solution was used for one second. Sections were stained with eosin for one minute, dehydrated in ethanol for five minutes, and cleared in xylene for another five minutes. Finally, the slides were covered with “Canada balsam” for light microscopical analysis.

The other seven positively charged slides, were studied immunohistochemically, with anti-TNF-a (Abcam, dilution 1:100) and anti-IL-6 (Santa Cruz, dilution 1:100) antibodies. The intensity of staining was evaluated by a semiquantitative scoring, based on a cross-count scale, according to the following categories: negative—no cross (–), mild—one cross (+), moderate—two crosses (++), intense—three crosses (+++). All slides were examined with brightfield microscopy by at least two independent observers, blinded to the identity of the immunohistochemical preparations [79].

#### 4.2.3. Preparation for Transmission Electron Microscopy

Retinal tissue samples were sectioned into <1 cm^3^ pieces. They were post fixed into glutaraldehyde 3% for 2 h, followed by 1 h into osmium tetroxide (OsO_4_) 1%. Staining was performed with uranyl acetate 1% for 16 h and then samples were dehydrated with ascending ethanol concentrations. Samples were embedded into Epon resin and ultra-thin sections (60–90 nm) were taken. Finally, sections were stained with Reynolds’s stain. Samples were observed under a TEM JEOL 1011 in 80 kV (JEOL-Tokyo, Tokyo, Japan).

#### 4.2.4. Statistical Analysis

For comparisons between groups, concerning the determined immunohistochemical intensity of IL-6 and TNF-a, the non-parametric Kruskal-Wallis test was employed. Within-group comparisons between IL-6 and TNF-a (valid only for group V; vide infra) were performed using the non-parametric Mann-Whitney U test. Level of significance was set at 5%. Data analysis was performed by use of the SPSS v.25 software (IBM Corp., Armonk, NY, USA).

### 4.3. Ethical Approval

The protocol number of the research’s statement on ethics approval and consent is 4.209/17-7-19 and was provided by the Committee for Bioethics and Ethics of Faculty of Medicine, Aristotle University of Thessaloniki.

## 5. Conclusions

In conclusion, in the present study, conventional histological techniques, such as eosin-hematoxylin staining do not demonstrate retinal lesions following intravitreal administration of voriconazole and micafungin. In contrast, electron microscopic investigation reveals ultrastructural lesions after both voriconazole as well as micafungin injection. The results of immunohistochemical stainings IL-6 and TNF-α are not indicative of any possible involvement of these two cytokines in the pathophysiology of retinal damage, possibly excluding the mild positive expression of IL-6 in intravitreal voriconazole infusion. The present research confirms and contributes to the existing literature as our findings are consistent with the findings of respective research protocols where the same dosages of drugs do not cause retinal damage (according to ERG and eosin-hematoxylin staining) making them safe to date but simultaneously offering a new perspective regarding the ultrastructural lesions. Histological retinal lesions after intravitreal injection of voriconazole and micafungin revealed with electron microscopy in the present protocol, raises the question of the safe usage of these antifungal agents in the treatment of fungal intraocular infections in the future.

The limitations of the present study open new perspectives for future investigation. The small number of laboratory animals and the absence of a repeated-dosing group suggest that further research should be conducted in order to extract a safe conclusion regarding the adverse effects of voriconazole and micafungin in the retina after intravitreal administration.
